# Utility of Teleorthodontics in Orthodontic Emergencies during the COVID-19 Pandemic: A Systematic Review

**DOI:** 10.3390/healthcare10061108

**Published:** 2022-06-14

**Authors:** Sabina Saccomanno, Vincenzo Quinzi, Arianna Albani, Nicola D’Andrea, Giuseppe Marzo, Guido Macchiarelli

**Affiliations:** Department of Health, Life and Environmental Science, University of L’Aquila, Piazza Salvatore Tommasi, 67100 L’Aquila, Italy; vincenzo.quinzi@univaq.it (V.Q.); arianna.albani@student.univaq.it (A.A.); dottnicoladandrea@gmail.com (N.D.); giuseppe.marzo@univaq.it (G.M.); guido.macchiarelli@cc.univaq.it (G.M.)

**Keywords:** COVID-19, teleorthodontics, fixed multibracket orthodontics, orthodontic urgencies, oral pain, oral care, gum swelling, orthodontic device, mucosal injuries, clear aligners

## Abstract

**Background.** Coronavirus disease has subjected the whole of humanity to two years of social isolation and a series of restrictions. These circumstances have led to the use of information technology in an increasingly widespread manner. Even in the dental field, telematic means have been used to respond to emergencies. The aim of this systematic review of the literature is to evaluate the types of orthodontic emergency that occurred most often and how they were managed by teleorthodontics during the COVID-19 pandemic. The secondary aim is that clinicians will use teleorthodontics not only during pandemics but as an additional tool to manage orthodontics. **Materials and Methods.** Out of 1695 articles available on PubMed, Science Direct, Cochrane and SciELO, eight articles were selected for this systematic literature review. Google Scholar was used as a secondary source to confirm that there were no additional articles. The screened papers comprised editorials, clinical studies, cross-sectional studies and retrospective studies in Italian, English or Spanish language. **Results.** The articles showed that the means by which patients most often communicated with their orthodontists were voice calls and smartphone applications such as WhatsApp^®^ Messenger. Through these media, patients communicated their orthodontic emergencies. These mainly involved fixed multibracket appliances and the most common issues were discomfort and pain, fracture or loss of the appliance, protruding distal ends of archwires, brackets, tubes and bands or retainer detachment. Through teleorthodontics, patients could solve these issues by using orthodontic relief wax, cutting the protruding distal ends of the archwire with a nail clipper or a stronger cutter and removing or replacing detached bands, brackets, tubes or metallic ligature with a clean tweezer. **Conclusions.** In situations where personal contact is limited, teleorthodontics represents a valuable aid for professionals and patients facing orthodontic emergencies. The hope is that it may continue to represent a valuable aid for patients with difficulties in planning an in-office visit.

## 1. Introduction

The coronavirus (COVID-19) epidemic is an ongoing, worldwide public health problem for which specific guidelines have been published, constantly updated by the World Health Organization (WHO). The competent ministries and regions directly or indirectly contribute to risk management through the identification of suspected cases and the activation of containment and quarantine measures for people who have had contact with suspected cases [1].

In the dental field, for the purpose of controlling COVID-19 infection, the fundamental preventive measure lies in the restriction of patients visiting the dental office [1].

It is universally acknowledged that dental treatments pose an extremely high risk of severe acute respiratory syndrome coronavirus infection because virus-contaminated aerosols could be generated potentially during the operation [2].

In the midst of the COVID-19 emergency, dental offices suspended all deferrable procedures in order to reduce the spread of the pathogen, although this was not always possible. Some ongoing treatments, such as fixed orthodontic therapies and/or conditions that must be identified and treated immediately to avoid more serious outcomes, require timely follow-up appointments. Indeed, continuous monitoring by the orthodontist is a requirement in orthodontic treatment so as to evaluate the efficacy and/or any undesirable effects. However, some periodic visits are not strictly necessary and others could be delayed by instructing the patient on how to make simple changes to the appliance—for example, by indicating on which teeth to place intraoral elastics or how many activations to perform on the central screw of a rapid palatal expander. Recently, an innovative approach has been imported from the medical into the dental field, namely telemedicine. Although it was originally developed to provide healthcare services in remote areas, it has proven to be essential in managing healthcare services in this unprecedented world-wide emergency situation. The World Health Organization (WHO) defines telemedicine as the use of telecommunications and virtual technologies to provide healthcare outside of traditional healthcare facilities. In more detail, telemedicine is a set of technologies, especially Information and Communication Technologies (ICT), specifically aimed at providing healthcare services from a distance to lessen the need for contact between the patient and the healthcare provider [3].

**Figure 1:**
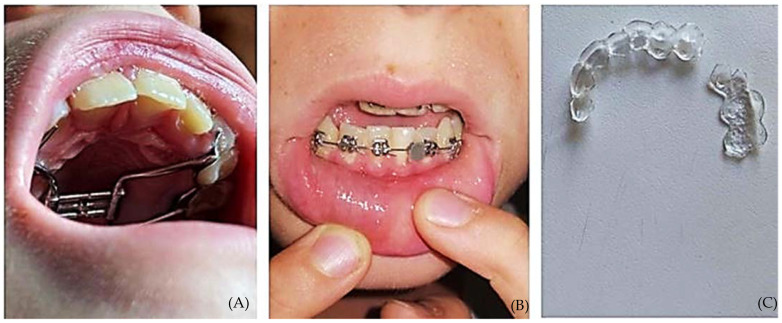
Problems usually reported with fixed or removable appliances: (**A**) gingival inflammation; (**B**) bracket detachment; (**C**) fractured aligners.

This is also a very important aspect in dentistry. Teledentistry is, in fact, a specialized extension of telemedicine that was first developed at the military level and subsequently found application mainly in the management of patients living in remote rural areas whose movements need to be limited to those necessary for reasons of greater fragility—for example, in the case of cancer patients. In the context of teledentistry, its application to orthodontics, called teleorthodontics, can be considered a very useful tool to assess patients remotely when they need to report a problem related to a fixed or removable device before a periodic check-up in person, or when they have queries about their usage (Figure 1) [4].

The tools used for teleorthodontics range from simple voice calls to the most advanced technologies. One example is described in an article by Thurzo et al., in 2021; they write that mobile orthodontic applications allow for the management of modern, aesthetic and comfortable treatments where patient compliance is key.

Dental Monitoring^®^ (DM) (Dental Monitoring Co., Paris, France) with remote monitoring is a current reality in orthodontics. Orthodontists can monitor patients with virtual examinations that supplement in-office appointments. Although this approach is hindered by negative connotations associated with the “direct to patient” business model, there are unquestionable benefits of remote monitoring for the clinical practice of orthodontics. The use of teleorthodontics such as DM has advanced the monitoring of patients during the COVID-19 situation. It has allowed clinicians to monitor all patients during the pandemic, reduced the costs and limited direct contact when it was not necessary. With all these means, it has decreased the risk of COVID-19 transmission and proved useful in addressing different clinical situations, such as loss of attachment, loss of various accessories, gingivitis, caries and many others [5].

An orthodontic urgency (OU) is defined as a problem related to orthodontic mechanics that requires rapid intervention to:

- Resolve severe physical discomfort (ulcers, irritation of the mucous membranes, lips or tongue);
- Treat infectious processes (periodontal abscesses associated with orthodontic device components);
- Manage problems related to orthodontic mechanics that could compromise the integrity of the dental support tissue or cause adverse effects if not controlled by an orthodontist;
- Reassure the patient in case of psychological problems.

The objective of this systematic review of the literature is to determine which types of orthodontic emergency occurred more often and how they were managed with teleorthodontics during the COVID-19 pandemic, in order to facilitate the use of teleorthodontics not only during a pandemic but also as an additional tool to manage orthodontic emergencies.

## 2. Materials and Methods

### 2.1. Guidelines

The PRISMA (Preferred Reporting Items for Systematic Reviews and Meta-Analysis) statement was adhered to as much as achievable [6].

### 2.2. PICO Question

This review was designed following PICO guidelines to determine the utility of teleorthodontics in managing orthodontic emergencies during the COVID-19 pandemic.

Population: patients facing orthodontic emergencies.

Intervention: use of teleorthodontics.

Comparison: orthodontic emergencies that can be managed through teleorthodontics versus orthodontic emergencies that cannot be managed through teleorthodontics.

Outcomes:

Primary outcome: teleorthodontics can be considered as a very useful tool to solve orthodontic urgencies when patients cannot attend in-office visits during the COVID-19 pandemic.

Secondary outcome: teleorthodontics can represent a valid aid not only during confinement, but also in different situations where patients cannot visit their orthodontist.

### 2.3. Search Strategy

The protocol of this research was registered in the publicly accessible PROSPERO (CRD42021229783), the primary database for registering systematic review protocols.

This systematic literature review was carried out up to November 2021 by searching for articles limited to humans with a publication range from December 2019 to November 2021 on five different search engines: PubMed, Science Direct, Cochrane and SciELO.

We used Google Scholar as a secondary source to confirm that there were no additional articles to those already included by searching through the aforementioned databases.

The following key phrases were entered in these scientific search engines: Teleorthodontics, Teledentistry and Orthodontics and Teleorthodontics Covid-19.

These key phrases were combined as follows:

(Teleorthodontics) or ((Teledentistry) and (Orthodontics)) and COVID-19

A manual search of the references of the chosen studies was performed.

### 2.4. Study Selection

The selection process was performed according to the following inclusion criteria:

- Included study designs: editorials, clinical studies, cross-sectional studies, retrospective studies;
- Articles evaluating the management of orthodontic emergencies during the COVID-19 pandemic;
- Articles performing studies on humans;
- Articles in English, Spanish and Italian languages;
- Articles with publication date from December 2019 to November 2021.

The selection process was then performed according to the following exclusion criteria:

- Excluded study design: case reports, comparative studies, bibliographic reviews, systematic reviews, meta-analyses, doctoral theses;
- Articles dealing with telemedicine or teleorthodontics not referring to the management of orthodontic emergencies during the COVID-19 pandemic;
- Articles carrying out studies on animals;
- Articles in languages other than English, Spanish and Italian;
- Articles with a publication date prior to December 2019.

### 2.5. Data Screening and Extraction

All the authors independently performed initial screening. At this stage, the title and abstract, if available, were investigated by all of the authors. The articles were selected if considered relevant by all the reviewers. A detailed full-text analysis was performed and four reviewers (S.S., V.Q., A.A. and N.D.A.) extracted data from each study. There were no dissentions related to data screening and extraction.

### 2.6. Data Analysis

This systematic review was carried out by using editorials, clinical studies, cross-sectional studies and retrospective studies and, once analyzed, we collected data regarding four areas:

- Instruments of teleorthodontics;
- Orthodontic emergency;
- Analyzed appliances;
- Resolution mode.

We based our work on these four groups of data collected from the selected articles.

Contrary to what was planned at registration on PROSPERO, we finally decided to include a longer time frame in the search for published articles (March 2020 to March 2021 vs. December 2019 to November 2021). This decision was made because the pandemic was protracted and we saw an increase in media, which we then wanted to examine in terms of their usefulness in the teleorthodontics field. To better examine the literature, we recruited an additional reviewer (G.M.)

### 2.7. Outcome Measures

The primary outcome was to evaluate whether teleorthodontics could be considered a useful tool to solve orthodontic emergencies when patients cannot attend in-office visits during the COVID-19 pandemic. The secondary outcome was to investigate teleorthodontics as a valid aid not only during confinement, but also in different situations where patients cannot visit their orthodontist.

### 2.8. Quality Assessment

Four reviewers (S.S., V.Q., A.A. and N.D.) independently evaluated the risk of bias. This evaluation was carried out following the Cochrane-recommended approach for assessing he risk of bias in randomized controlled clinical studies, including four quality parameters: sequence generation, consideration of incomplete outcome data, freedom of selective outcome reporting and other sources of bias [7].

### 2.9. Levels of Evidence

Levels of evidence and grade of recommendation followed the Grading Recommendations of the GRADE Working Group [8].

Our eight final studies, published in 2020 and 2021, were all level 2B since we excluded reviews and meta-analyses, which would have been level 1A.

## 3. Results

### 3.1. Study Selection

A total of 1695 articles were available from the first search. By entering the filters specified in the inclusion criteria in the five search engines, 751 articles were excluded.

**Figure 2:**
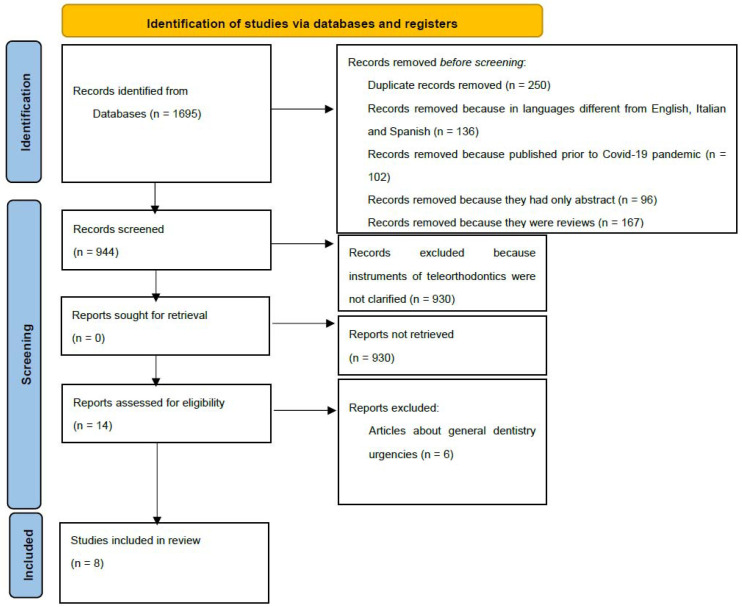
Identification of studies via databases and registers.

Of the remaining 944 articles, after reading the abstracts, including duplicates (same article in common for different key phrases used), 930 were excluded. Therefore, 14 articles were read, of which six were excluded because, although they dealt with the use of teleorthodontics during the COVID-19 pandemic, they did not mention the management of orthodontic emergencies. Eight articles were analyzed for this systematic literature review, and Figure 2 shows the process used in this review, which was based on the PRISMA guidelines for systematic reviews.

### 3.2. Assessment of Risk of Bias

The studies were graded as having high, uncertain or low risk, based on selection bias, performance bias, detection bias, attrition bias and reporting bias. The quality of individual studies was evaluated based on the categorized ranking of the Oxford Centre for Evidence-Based Medicine 2011 Levels of Evidence.

### 3.3. Effects of Interventions

All authors subdivided the possible orthodontic emergencies according to the devices used to treat the patients, except for the study by Jie Xiang et al., in which only patients treated with fixed multibracket orthodontics were studied.

Caprioglio et al. and Matus et al. managed orthodontic emergencies via teleorthodontics (email, phone call, Skype, Google Duo or Zoom), initially gathering information about the patient’s health status—in particular, symptoms and relevant antecedents with respect to a possible COVID-19 infection. In a second step, they guided the patient to the resolution of the emergency by themselves, interrupting therapy momentarily if necessary. In cases where this was not possible, an emergency visit was organized, ensuring that the dental office met the necessary requirements to avoid possible transmission of COVID-19 [1,9].

Xiang et al., in their study of 617 orthodontic patients at West China Hospital, who were unable to undergo regular orthodontic checkups, reported that 418 of these patients did not seek any OU care, despite not being able to be seen frequently due to the pandemic. Instead, they recorded the most frequent orthodontic emergencies in 199 patients who came to the hospital to be seen [2].

Putrino et al. evaluated the efficacy of using teleorthodontics through well-defined clinical instructions using the video call service of the WhatsApp^®^ mobile phone application. Again, data points were broken down according to the device with which patients were treated, and the most frequent emergencies that occurred for each device were identified. Unlike in the other studies, the follow-up of patients undergoing treatment was evaluated, rather than the management of emergencies by teleorthodontics [4]. 

Questionnaires were used in the remaining four articles.

Arqub et al., in their study of 154 patients in the Department of Orthodontics at Connecticut Health University, found that only 21% of these had OU. Orthodontic monitoring and management of possible OU by teleorthodontics—in this case, phone call or message—was preferred by 73% of patients, avoiding visits in person during the pandemic [10].

Bustati et al. evaluated the experiences of 388 patients undergoing orthodontic treatment. In addition, in this study, emergencies were classified according to the type of orthodontic device used. In particular, 38% of patients who were undergoing treatment with fixed orthodontics, 18% of patients undergoing treatment with removable devices and 57% of patients undergoing treatment with clear aligners contacted the orthodontist via telephone call for OU resolution. Meanwhile, 13% of patients who were being treated with fixed orthodontics and 12% of patients who were being treated with removable devices contacted the orthodontist by sending a photo from their cell phone [11].

Saccomanno et al. also evaluated the experiences of 30 orthodontic patients monitored via teleorthodontics (video calls, dedicated apps, photo sharing or instant messaging), treated with different devices: multibrackets orthodontics, orthodontic aligners, palate expanders and functional mobile devices. They created a system of new emojis, an easy technique to involve younger and shy patients by enabling them to describe their experience regarding the follow-up orthodontic treatment and the management of possible emergencies through telorthodontics [3].

Moreover, in the study of Cotrin et al., questionnaires were used, which, unlike in the three previous studies, were completed by orthodontists from four different Brazilian universities. The study identified the most frequent orthodontic emergencies and the device that most frequently required intervention by the orthodontist via teleorthodontics (WhatsApp^®^ or telephone call) [12].

The orthodontic devices used to treat the patients included in the above articles and the most frequent OUs are specified in Table 1 of the results.

The types of instruments used for teleorthodontics and the resolution mode based on the type of emergency are specified in Table 2.

## 4. Discussion

### 4.1. Overall Discussion

The emergency situation caused by the new coronavirus has posed many challenges for governments and countries. Efforts to limit its prevalence and, of course, its socioeconomic and therapeutic costs have forced governments to impose temporary regulations (lockdowns) to reduce social interactions. The dental field, because of its high potential for transmission, has been severely affected by the strict regulations imposed by organizations and committees responsible for limiting the spread of the new coronavirus [1].

Being at a high risk level of infection, most dental clinics were closed during the various lockdowns, which placed patients who were receiving orthodontic treatment in a complicated situation, since they typically require regular visits to their orthodontist for a long period of time [11].

Thousands of orthodontic patients in lockdown areas missed their monthly visits because of the long duration of the orthodontic treatment and its elective nature. The sudden halting of treatment and possible problems that orthodontic appliance components created over this long period caused great concern and confusion for both patients and orthodontists. Although orthodontic treatment has esthetic and elective aspects, this does not mean that no emergency condition will occur [1]. 

Telemedicine, from which teledentistry originates—and, by definition, teleorthodontics—although not particularly widespread as a diagnosis and control strategy and reserved only for certain categories of patients and situations, can be a useful tool by which to maintain contact with a patient under orthodontic therapy [4]. 

The articles reviewed showed that the means by which patients most often communicated with their orthodontists were:

1. Voice calls [1,2,9,10,11];
2. Smartphone applications such as WhatsApp^®^ Messenger [1,4,9,10,11,12].

This is because recent studies showed that smartphones provided fast and clear access to electronically mailed digital images and allowed professionals free mobility, not restricted by the constraints of a desktop personal computer [12]. 

Through these media, patients were able to communicate their orthodontic emergencies. These mainly involved fixed multibracket appliances with the following issues:

1. Detachment of brackets, tubes or bands [1,2,4,9,10,11,12];
2. Protruding arch ends [1,2,9,10,11];
3. Loss or displacement of metal or elastic bindings [1,4,11,12].

In the case of removable devices or clear aligners, however, it was mostly a matter of loss or breakage of the device. 

Depending on the case, teleorthodontics allowed orthodontists, within the limits of their possibilities, to solve several orthodontic emergencies. 

In the case of fixed multibracket devices, the indication has been to cover the protruding areas that cause ulcers and soft tissue lesions with orthodontic wax [1,4,11] or, where this is not possible, the recommended options have been the following:

1. Cut off the protruding arch ends [1];
2. Bend the ends of the protruding metal ligatures or remove the ligatures with clean tweezers [1];
3. Remove detached tubes, bands and brackets with clean tweezers where possible, if there is a risk of accidental ingestion [1].

Conditions that necessitate an emergency orthodontic examination in person are those in which symptoms do not regress by waxing or cutting the arch, and cases in which the mobility of metal parts places the patient at risk of accidental ingestion [9]. 

Regarding orthodontic emergencies in patients using removable devices and invisible aligners, resolution of situations involving broken and lost devices was achieved by recommending discontinuation of therapy in the case of removable devices [1,4] or wearing the predecessor or subsequent template in the case of invisible aligner therapy [1].

In addition to this, Saccomanno et al. state that problems such as the poor fit of aligners could be easily solved by teleorthodontics by suggesting that patients place the masks in slightly warm water [3].

Teleorthodontics, in the management of emergencies during the pandemic, was appreciated by both clinicians and patients, and the articles reviewed in this review highlight multiple benefits. These include the reduced risk of infection; the ability to triage remotely to see if the patient needs an office visit or if there is an opportunity to resolve the problem through the media [9,10,11]; the reduction in the time the patient spends in the chair [3]; and the ability to quickly send and evaluate images and radiographs [1,12]. In addition, other positive aspects should be emphasized, such as being able to reassure patients who perceive anxiety, fear and a sense of abandonment during therapy [1,3,4], and the possibility of periodically monitoring the oral health status of patients, motivating them to maintain a good level of hygiene [3,4,10].

On the other hand, disadvantages associated with teleorthodontics were also examined. 

Saccomanno et al., in their 2020 article, consider the use of teledentistry a good solution, even if it does not seem to be applicable in every situation and for long periods of time. Clinicians showed that only functional appliances and aligners (such as Invisalign^®^) could be managed for a long time through the use of teleorthodontics, needing only follow-up to continue therapy. On the other hand, the progression of multibracket treatment is limited in teledentistry, because of its intense hands-on characteristic. Almost all the required follow-ups need in-office visits, except for occasional checkups on oral hygiene or to solve an issue [3].

Putrino et al., in their study, also discuss the disadvantages and assert that it is difficult to replace the evaluations carried out during in-person objective clinical examinations with teleorthodontic checks. Furthermore, remotely viewing radiographs such as orthopanoramics, lateral teleradiographs or more accurate examinations such as TC DentaScan or CBCT may be helpful in difficult initial consultations to assess the need for complex treatments such as extractions or evaluations on the importance of certain radiographic elements. In addition to this, the authors believe that the standardization of protocols for teleorthodontics would make this method very useful in following up with patients unable to attend regular follow-ups, such as those who are disabled, sick, frequent travelers or residing in rural areas distant from the dental office [4].

In the study by Arqub et al., the results of their questionnaire administered to orthodontic patients showed that the majority of patients declared themselves neutral towards the use of teleorthodontics, but they appreciated the possibility of telephone triage before scheduling any visit [10].

Another scenario is advancing in the field of teleorthodontics, namely orthodontic smart applications. In the 2020 work by Saccomanno et al., in addition to video calls and instant messaging, the authors mention an application called *Smile Consult* by Align Technology Inc., used to monitor the progress in occlusion and compare it with the ClinCheck simulated progress provided by the company. This application gives the possibility for orthodontists to provide web meetings and offer quick responses to patients’ issues. It still allows for traditional appointments, in addition to easily scheduling appointments online, if and when patients have their own camera to share photos of the mouth and the teeth [3].

Thurzo et al. also focus attention on smart apps to monitor patients remotely and state that dentistry needs to expand its understanding of how dental apps, digital workflow models and digital health information are transforming dental practice in order to predict how this digital shift will impact the entire field of dentistry [5].

Since technology is constantly evolving, these applications may soon be implemented so that they can be used in routine orthodontist–patient communication.

In addition to the contribution that this work may make to the literature, the goal is to improve the daily routine of orthodontists and patients. If, during the pandemic, teleorthodontics has been very useful in solving orthodontic problems and reducing contagion, similarly, it could become very useful in general practice, with technologies that will become increasingly accurate.

In light of what has emerged from the review of the articles, it is evident that teleorthodontics can be considered a valuable aid in the management of orthodontic emergencies when there is no opportunity to visit patients in person. Depending on the emergency and the equipment used, the clinician can monitor patients and instruct them on how to solve a problem without subjecting them to a visit, while reducing the anxiety and discomfort that may be perceived. 

We believe that this method of remote monitoring can also be used in current daily practice to reduce the time and costs that result from periodic check-ups, to ascertain the level of oral hygiene and thus the health of the oral cavity of the patients and to enable accessible screening, not only in orthodontics but in the dental field in general, benefitting patients who:

1. Live far from population centers and have few possibilities to reach hospitals or private practices;
2. Are disabled or ill;
3. Are students of preschool, primary and secondary school, to keep their oral hygiene under control and intercept orthodontic problems;
4. Are living in conditions of socio-economic disadvantage;
5. Live temporarily abroad and have no way to return for regular check-ups;
6. Are undergoing follow-up after the end of orthodontic treatment.

Nonetheless, it is necessary to emphasize the importance of preventing orthodontic emergencies, as was also mentioned in the article by Bustati et al., to avoid patients experiencing problems that may require emergency visits, or, in the worst cases, even having to go to the emergency department at the hospital. Bustati and colleagues refer professionals to *the British Orthodontic Society COVID-19 Orthodontic Emergency Protocol*, but, more generally, they point to the fact that patients should be trained to manage these problems whenever possible. In addition, according to the same authors, orthodontists can also benefit from the many recommendations and guidelines that have been suggested already in the literature, to allow them to provide their patients with proper care while protecting themselves as well [11].

However, it is emphasized that, within the limits of use described above, teleorthodontics should be implemented and would need further standardization in order to allow the analysis of data from the patients and telecommunicate with them safely and with respect for privacy, always after signing a detailed informed consent form to be submitted to the patients or their parents/guardians. 

### 4.2. Study Limitations

The major limitation of this study lies in the small number of articles found using the search engines. For this reason, we considered it appropriate to increase our search time until November 2021. In this way, we were able to obtain the final eight articles. 

In addition to this, we did not detect homogeneous studies and therefore a meta-analysis could not be performed.

Moreover, it should be mentioned that our topic has, as its background, the pandemic, which is an ongoing event. As the pandemic progresses, technology is also evolving, and what we find useful today may be obsolete before long.

In summary, we can assert that our systematic review provides a snapshot of this historical period (December 2019 to November 2021).

## 5. Conclusions

We can assert that when personal contact is limited, as in the recent case of the pandemic, teleorthodontics represents a valuable aid for professionals and patients facing orthodontic emergencies. Some of these can be resolved remotely, while others require an emergency visit. With a pre-triage, patients are categorized into one of two options, and emergencies that do not require a visit can be resolved by providing the appropriate indications, depending on the type of equipment and emergency.

The hope of the authors is that teleorthodontics may continue to represent a valuable aid for patients with difficulties in attending an in-office visit.

## Figures and Tables

**Table 1:** Results.

Title	Year	Authors	Analyzed Appliances	Orthodontic Emergency
Management of orthodontic emergencies during 2019-NCOV [1]	2020	Caprioglio et al.	1. Fixed multibrackets 2. Removable (functional and aligners)	1. Fracture or loss 2. Injury to lips or cheek 3. Bracket, tube, band or retainer detachment
Perspectives of tele-orthodontics in the COVID-19 emergency and as a future tool in daily practice [3]	2020	Saccomanno et al.	1. Fixed multibrackets 2. Fixed (i.e., maxillary expander) 3. Orthodontic aligners 4. Removable	1. Gingival inflammation 2. Orthodontic aligner deformation
Appointment impact and orthodontic emergency occurrence during the coronavirus disease 2019 pandemic: A retrospective study [2]	2021	Jie Xiang et al.	1. Fixed multibracket	1. Bracket or band detachment 2. Excess arch in the distal ends 3. Arch fracture 4. Lingual button detachment 5. Loss of coil spring/power chain or anchorage device 6. Dysfunction of pre-activated devices and removable accessory devices 7. Loss of metal ligature 8. Periodontal, endodontic and mucosal symptoms
Impact of the SARS-Cov2 pandemic on orthodontic therapies: an Italian experience of Teleorthodontics [4]	2020	Alessandra Putrino et al.	1. Fixed multibrackets 2. Removable 3. Orthodontic aligners	1. Bracket detachment 2. Loss of ligatures 3. Discomfort and pain
Atención de Pacientes en Tratamiento de Ortodoncia Durante la Pandemia COVID-19 (SARS-CoV-2). Presentación de un algoritmo [9]	2020	Matus A. et al.	1. Fixed appliances	1. Pain or inflammation 2. Detachment of device, bracket, band or tube 3. Dentoalveolar trauma 4. Mucosal injury 5. Complications due to a recent surgical procedure, related to orthodontic treatment
Patients’ perceptions of orthodontic treatment experience during COVID-19: a cross-sectional study [10]	2021	Sarah Abu Arqub et al.	1. Fixed multibracket	1. Bracket detachment 2. Mucosal lesions caused by the distal portion of the arch
The impact of COVID-19 pandemic on patients receiving orthodontic treatment. An online questionnaire cross sectional study [11]	2020	Nour Bustati et al.	1. Fixed multibracket 2. Removable 3. Orthodontic aligners	1. Bracket or band detachment 2. Excessive arch in distal ends 3. Loss or mismatch of elastics 4. Loss of device 5. Pain related to orthodontic movement 6. Appearance of spaces 7. Fracture of device parts
Urgencies and emergencies in orthodontics during the coronavirus disease 2019 pandemic: Brazilian orthodontists’ experience [12]	2020	Paula Cotrin et al.	1. Fixed multibracket 2 Fixed (i.e., maxillary expander) 3. Orthodontic aligners 4. Removable	1. Detachment of brackets, arch, tubes, bands, retainer or maxillary expander 2. Sharp metal ligature 3. Loss of elastic ligature 4. Poor oral hygiene 5. Fractured aligners or removable devices

**Table 2:** Instruments of teleorthodontics and resolution mode.

Title	Instruments of Teleorthodontics	Orthodontic Emergency	Resolution Mode
Management of orthodontic emergencies during 2019-NCOV [1]	1. Whatsapp^®^ Messenger	1. Fracture or loss 2. Injury to lips or cheeks 3. Bracket, tube, band or retainer detachment 4. Protruding distal ends of archwire 5. Elastic ligature mismatch 6. Loss or breakage of aligners	1. Orthodontic relief wax 2. Suspend using broken or non-fitting removable appliances 3. Cut protruding distal ends of the archwire with a nail clipper or a stronger cutter 4. Remove or replace detached bands, brackets, tubes or metallic ligature with a clean tweezer 5. If resolution is not possible, an emergency visit is required
Perspectives of tele-orthodontics in the COVID-19 emergency and as a future tool in daily practice [3]	1. Video calls (Zoom Video Communications, Inc.) 2. A dedicated application (Smile Consult by Align Technology Inc., San Jose, CA, USA) 3. Instant messaging	1. Gingival inflammation 2. Orthodontic aligner deformation	1. Place non-fitting aligners in mild hot water
Impact of the SARS-Cov2 pandemic on orthodontic therapies: An Italian experience of Teleorthodontics [4]	1. Phone calls 2. Video calls	1. Bracket detachment 2. Loss of ligature 3. Discomfort and pain	1. Suspend using broken removable appliances 2. Orthodontic relief wax
Atención de Pacientes en Tratamiento de Ortodoncia Durante la Pandemia COVID-19 (SARS-CoV-2). Presentación de un Algoritmo [9]	1. Phone calls 2. Emails 3. WhatsApp^®^ Messenger	1. Pain or inflammation 2. Detachment of device, brackets, band or tube 3. Dentoalveolar trauma 4. Mucosal injury 5. Complications due to a recent surgical procedure, related to orthodontic treatment	1. Indications via teleorthodontics 2. Planning in-office visit if emergency cannot be remotely solved
The impact of COVID-19 pandemic on patients receiving orthodontic treat-ment. An online questionnaire cross sectional study [11]	1. Phone calls 2. Smartphone applications	1. Bracket or band detachment 2. Excessive arch in distal ends 3. Loss or mismatch of elastics 4. Loss of device 5. Pain related to orthodontic movement 6. Appearance of spaces 7. Fracture of device parts 8. Gum swelling	1. Orthodontic relief wax
